# A scoping review of the Clinical Frailty Scale

**DOI:** 10.1186/s12877-020-01801-7

**Published:** 2020-10-07

**Authors:** Sophie Church, Emily Rogers, Kenneth Rockwood, Olga Theou

**Affiliations:** 1 Department of Medicine, Dalhousie University, Halifax, Nova Scotia Canada; 2 Department of Psychiatry, Dalhousie University, Halifax, Nova Scotia Canada; 3 Division of Geriatric Medicine, Queen Elizabeth II Health Sciences Centre, Nova Scotia Health, Halifax, Nova Scotia Canada; 4 School of Physiotherapy, Dalhousie University, Halifax, Nova Scotia Canada

**Keywords:** Frailty, Aging, Frail elderly, Scoping review, Clinical Frailty Scale

## Abstract

**Background:**

Frailty is increasingly recognized as an important construct which has health implications for older adults. The Clinical Frailty Scale (CFS) is a judgement-based frailty tool that evaluates specific domains including comorbidity, function, and cognition to generate a frailty score ranging from 1 (very fit) to 9 (terminally ill). The aim of this scoping review is to identify and document the nature and extent of research evidence related to the CFS.

**Methods:**

We performed a comprehensive literature search to identify original studies that used the Clinical Frailty Scale. Medline OVID, Scopus, Web of Science, CINAHL, PsycINFO, Cochrane Library and Embase were searched from January 2005 to March 2017. Articles were screened by two independent reviewers. Data extracted included publication date, setting, demographics, purpose of CFS assessment, and outcomes associated with CFS score.

**Results:**

Our search yielded 1688 articles of which 183 studies were included. Overall, 62% of studies were conducted after 2015 and 63% of the studies measured the CFS in hospitalized patients. The association of the CFS with an outcome was examined 526 times; CFS was predictive in 74% of the cases. Mortality was the most common outcome examined with CFS being predictive 87% of the time. CFS was associated with comorbidity 73% of the time, complications 100%, length of stay 75%, falls 71%, cognition 94%, and function 91%. The CFS was associated with other frailty scores 94% of the time.

**Conclusions:**

This scoping review revealed that the CFS has been widely used in multiple settings. The association of CFS score with clinical outcomes highlights its utility in the care of the aging population.

## Background

Worldwide, the population of older adults is expected to grow from 8.5% of people aged 65 and older to nearly 25% by 2050 [1]. As the population ages, the concept of frailty becomes increasingly relevant in the provision of health care to the population. Frailty was introduced nearly three decades ago to the geriatric medicine literature as a method of understanding and discerning the complex health status of older adults. Frailty is defined as a state in which there is an increase in an individual’s vulnerability for developing increased dependency and/or mortality when exposed to a physiological or psychological stressor [2]. Frailty can also be thought of as tipping the balance between reserve and insults, or positive and negative influences, leading to increased vulnerability [3]. People living with frailty are more vulnerable to the effects of potential stressors, and to deterioration than others of the same chronological age.

Frail individuals experience higher rates of adverse outcomes [4]. As such, assessing frailty early in the course of care is essential in order to identify those patients who are most vulnerable. Frailty measurement is integral for guiding patient care, as it helps clinicians determine which interventions will be more likely to be beneficial and which may be more harmful to particular individuals [5], for example more aggressive medical treatments. It has been suggested that all individuals over the age of 70 be screened for frailty [6]. Despite agreement on it being a multiply determined state of increased risk, consensus on a frailty definition has yet to be reached.

The Clinical Frailty Scale is a clinical judgement-based frailty tool developed for the Canadian Study of Health and Aging [7]. There it summarized the results of a Comprehensive Geriatric Assessment facilitating discussion of the impact of frailty when assessments had been conducted by physicians and nurses from a range of disciplines. The CFS evaluates specific domains including comorbidity, function, and cognition to generate a frailty score ranging from 1 (very fit) to 9 (terminally ill). Various reviews have been published on frailty suggesting the CFS is a promising frailty screening tool [8–10], however none of these have focused specifically on the CFS. Although the CFS is used commonly in both research and clinical care, there is currently no synthesis of its use. The objective of this scoping review was to map and synthesize the literature around the use of the CFS. This included identifying and documenting the nature and extent of research evidence pertaining to the CFS, with the aim of providing a more comprehensive description of its use across settings. We also examined the ability of the CFS to predict adverse health outcomes. This knowledge could inform and support clinicians and policy makers in making decisions about resource allocation and service access.

## Methods

### Search strategy

The search strategy for this scoping review was developed with assistance from a librarian, with the aim of identifying all original studies that assessed the Clinical Frailty Scale (Appendix 1). A variety of databases including Medline OVID, SCOPUS, Web of Science, CINAHL, PsycINFO, Cochrane Library and Embase were searched from 2005 when the original CFS paper was published to March 2017. All relevant articles retrieved from this search strategy were included for screening. Additional studies were identified by manually searching the reference lists of potentially relevant papers and other frailty systematic reviews. SCOPUS and Web of Science searches were also completed for all articles citing the article which described the development of the CFS tool [7]. All articles identified were imported into *Covidence* software for screening.

### Inclusion/exclusion criteria

Studies were only included when 1) they were original research and 2) the CFS was used. Due to the broad nature of this scoping review, we did not limit by language, study design, setting, age of participants or outcome measure. For non-English articles, members of the research team or colleagues with knowledge of the given language screened them and assisted in data extraction. Quality assessment is not a priority in scoping reviews; thus, studies were not excluded based on their quality.

### Title and abstract screening

Title and abstract screening was completed in *Covidence* software. The only inclusion criterion for the title and abstract screening was whether the article described an original study. Review articles, conference abstracts, editorials and commentaries were excluded from the review. Screening was completed by two independent reviewers and conflicts were managed and resolved by discussion between the two reviewers, including a third reviewer when necessary.

### Full text screening

Full text screening was also completed in *Covidence* software. Studies that include the Clinical Frailty Scale in their methods were included for data extraction; protocol papers proposing the use of the CFS in a study were also included. Studies that reference the CFS but did not assess or plan to assess frailty with the CFS in a group of participants were excluded. Full text screening was completed by two independent reviewers, with conflicts managed by discussion to achieve consensus or by a third reviewer when necessary.

### Data extraction and analysis

A data extraction form on Microsoft Excel was used to guide the collection of information from each article. Data was first extracted from 10 articles, and then this form was modified before the completion of the data extraction. The following descriptive data was extracted from each article that satisfied the inclusion criteria: year of publication, language, country, study design, and study setting. For completed studies (not protocol studies) participant demographics were extracted, including number of participants, and participant age and sex. With respect to the CFS, we extracted information about persons who administered the CFS, cut points for identification of frailty, frailty prevalence, and the purpose of the CFS assessment. All outcomes and variables that studies assessed for a relationship with CFS were recorded. As numerous outcome variables were identified, outcomes were grouped according to overarching themes for the purpose of analysis. Analyses were conducted using IBM SPSS 21.

## Results

**Fig. 1:**
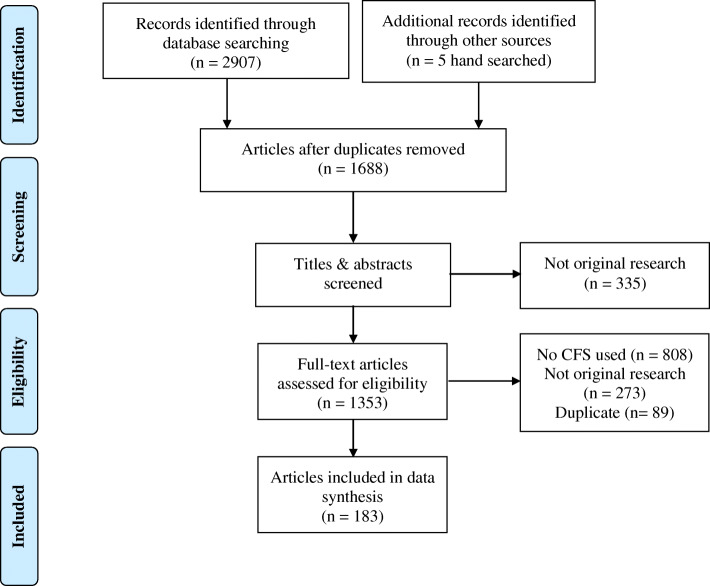
Scoping review flow chart

**Fig. 2:**
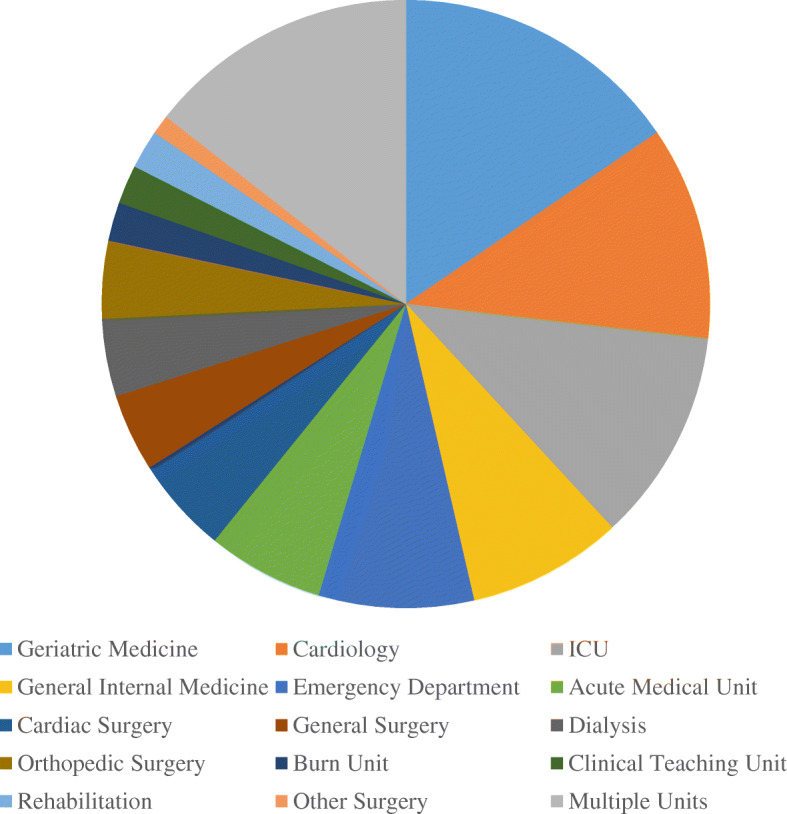
Proportion of studies that took place on various hospital units/wards

Our database search retrieved 2907 articles and hand-searching identified 5 additional articles for a total of 2912. After duplicates were removed, 1688 articles were included for title and abstract screening. We screened the full text of 1353 articles and 183 articles (Appendix 2) were identified for data extraction (Fig. 1). The CFS was measured in hospitalized patients in 63% of the articles and in the community in 18% of the articles. Of the 97 in-hospital studies that reported the unit or ward, the most common units were Geriatric Medicine (15%) and Cardiology (11%) (Fig. 2). Of the 183 included studies, 168 were written in English (92%), most studies were conducted in Canada (29%) and in the United Kingdom (21%), and 62% of studies were conducted after 2015 (Table 1). In relation to study design, 85% of studies were observational and 14% were experimental; of these 25 experimental studies, 18 were randomized controlled trials (Table 1).

**Table 1:** Study characteristics stratified by study setting

	All	Hospital	Long-term Care	Outpatient Clinic	Community	Hospital and Community
Articles n (%)	183 (100)	115 (63.2)	10 (5.5)	13 (7.1)	32 (17.6)	12 (6.6)
Year n (%)						
2015+	114 (62.2)	80 (69.6)	4 (40.0)	10 (76.9)	11 (34.4)	9 (75.0)
2010–2014	63 (34.4)	34 (29.6)	5 (50.0)	3 (23.1)	19 (59.4)	2 (16.7)
2005–2009	6 (3.3)	1 (0.9)	1 (10.0)	0	2 (6.3)	1 (8.3)
Language n (%)						
English	168 (91.8)	103 (89.6)	9 (90.0)	13 (100)	31 (96.9)	11 (91.7)
German	5 (2.7)	5 (4.3)	0	0	0	0
Spanish	2 (1.1)	1 (0.9)	0	0	0	0
Japanese	3 (1.6)	2 (1.7)	0	0	0	1 (8.3)
Italian	2 (1.1)	2 (1.7)	0	0	0	0
Polish	2 (1.1)	1 (0.9)	0	0	1 (3.1)	0
Cantonese	1 (0.5)	1 (0.9)	0	0	0	0
Country n (%)						
Canada	53 (28.9)	32 (27.8)	3 (30.0)	4 (30.8)	8 (25.0)	6 (50.0)
UK	39 (21.3)	30 (26.1)	2 (20.0)	2 (15.4)	2 (6.3)	3 (25.0)
USA	6 (3.3)	4 (3.5)	0	0	1 (3.1)	0
Australia	10 (5.5)	8 (7.0)	0	0	2 (6.3)	0
Germany	10 (5.5)	7 (6.1)	0	2 (15.4)	1 (3.1)	0
Italy	6 (3.3)	5 (4.3)	0	1 (7.7)	0	0
Ireland	9 (4.9)	3 (2.6)	0	0	6 (18.8)	0
Taiwan	8 (4.4)	3 (2.6)	0	0	5 (15.6)	0
Japan	8 (4.4)	3 (2.6)	2 (20.0)	1 (7.7)	1 (3.1)	1 (8.3)
Poland	6 (3.3)	4 (3.5)	1 (10.0)	0	1 (3.1)	0
Netherlands	4 (2.2)	2 (1.7)	0	1 (7.7)	1 (3.1)	0
China	3 (1.6)	1 (0.9)	1 (10.0)	0	1 (3.1)	0
Colombia	2 (1.1)	2 (1.7)	0	0	0	0
Spain	2 (1.1)	1 (0.9)	1 (10.0)	0	0	0
Singapore	2 (1.1)	1 (0.9)	0	0	1 (3.1)	0
Other^a^	7 (3.5)	4 (3.6)	0	1 (7.7)	1 (3.1)	1 (8.3)
Multiple countries	3 (1.6)	1 (0.9)	0	1 (7.7)	0	1 (8.3)
Number of participants						
# of articles reporting	177	110	9	13	32	12
Range	1–27,527	1–12,282	15–728	32–3500	1–27,527	7–2305
Median (IQR)	261.0 (100.0–548.5)	305.0 (101.0–530.0)	160.0 (93.0–279.0)	124.0 (86.0–1023.5)	261.5 (95.3–803.0)	164.0 (44.0–525.0)
Mean age of participants						
# of articles reporting	135	84	7	9	24	11
Range	54.0–93.0	55.6–93.0	82.0–90.9	72.0–85.4	56.68–87.2	54.0–84.4
Median (IQR)	79.0 (71.4–82.9)	79.8 (70.8–82.9)	85.5 (82.9–88.0)	81.3 (75.4–83.0)	76.0 (70.9–79.5)	79.0 (62.0–84.2)
Percentage of females						
# of articles reporting	150	91	8	12	27	12
Range	0.0–100.0	0.0–90.7	64.0–88.8	23.5–77.8	32.0–100.0	29.0–100.0
Median (IQR)	54.5 (44.0–64.0)	50.6 (41.8–57.0)	77.5 (74.0–79.6)	55.9 (37.8–74.1)	58.6 (52.5–64.0)	59.6 (50.3–68.8)
Study design n (%)						
Observational	156 (85.2)	101 (64.7)	10 (6.4)	11 (7.1)	25 (16.0)	9 (5.8)
Experimental	25 (13.7)	12 (48.0)	2 (8.0)	2 (8.0)	6 (24.0)	3 (12.0)
Qualitative	2 (1.1)	1 (50.0)	0	0	1 (50.0)	0

Note that as one article did not specify setting, it is only included in the “All” column

^a^Other countries included New Zealand, Finland, Sweden, Brazil, Austria, France and Lithuania

# number; *IQR* interquartile range

The CFS was assessed by researchers (46% of articles) and by a variety of professionals with physicians being the most common (24%). The CFS was originally developed as a 7-point scale but there is currently an updated 9-point version. Among the included articles, 58% used the 7-point scale and 38% used the 9-point scale (Table 2) with the 9-point version being the most common among the most recent studies (Supplementary Figure. 1). When categorized, a CFS score of five was the most widely used frailty cut point (68.9%). Most studies (46.4%) used the CFS for risk stratification (i.e. to predict outcomes) (Table 2, Supplementary Figure 2). Of the 31 times the association between CFS score and age was examined, 77% found a significant relationship. Female sex was correlated with CFS score in 50% of the 18 times this association was tested (Supplementary Table 1).

**Table 2:** Frailty characteristics stratified by setting

	All	Hospital	Long-term Care	Outpatient Clinic	Community	Hospital and Community
Articles n (%)	183 (100.0)	115 (63.2)	10 (5.5)	13 (7.1)	32 (17.6)	12 (6.6)
Site of CFS assessment n (%)						
# articles reporting	180	112	10	13	32	12
On site	137 (76.1)	85 (75.9)	8 (90.0)	10 (76.9)	24 (75.0)	9 (75.0)
Retrospectively	27 (15.0)	18 (16.1)	1 (10.0)	2 (15.4)	4 (12.5)	2 (16.7)
On-site & retrospective	13 (7.2)	9 (8.0)	1 (10.0)	1 (7.7)	1 (3.2)	1 (8.3)
Telephone	3 (1.7)	0	0	0	3 (9.4)	0
Times CFS assessed n (%)						
Once	173 (94.5)	110 (95.6)	10 (100.0)	11 (84.6)	29 (90.6)	12 (100.0)
Twice	8 (80.0)	3 (33.3)	0	2 (20.0)	3 (33.3)	0
Three times	1 (10.0)	1 (10.0)	0	0	0	0
Four times	1 (10.0)	1 (10.0)	0	0	0	0
Person administering CFS n (%)						
# articles reporting	111	69	3	7	24	8
Researchers	51 (45.9)	33 (47.8)	0	1 (14.3)	13 (54.1)	4 (50.0)
Physicians	27 (24.3)	17 (24.6)	1 (10.0)	1 (14.3)	5 (20.8)	3 (37.5)
Nurse/NP	11 (9.9)	8 (121.6)	0	0	3 (12.5)	0
Other staff^a^	4 (3.6)	1 (0.9)	1 (10.0)	0	1 (4.2)	1 (12.5)
Clinical staff not reported	4 (3.6)	1 (0.9)	1 (10.0)	2 (28.6)	0	0
Mixed	14 (12.6)	9 (13.0)	0	3 (42.9)	1 (4.2)	0
Version of CFS used n (%)						
# articles reporting	153	96	9	9	30	9
7-point	89 (58.2)	48 (50.0)	5 (55.6)	7 (77.8)	22 (73.3)	7 (77.8)
8-point	6 (3.9)	5 (5.2)	0	0	1 (3.3)	0
9-point	58 (37.9)	43 (44.8)	4 (44.4)	2 (22.2)	7 (23.3)	9 (22.2)
Frailty cut-point n (%)						
# articles reporting	58	42	1	4	9	2
3	1 (1.7)	1 (2.4)	0	0	0	0
4	15 (25.9)	10 (23.8)	0	3 (75.0)	2 (22.2)	0
5	40 (68.9)	29 (69.0)	1 (100.0)	1 (25.0)	7 (77.8)	2 (100.0)
6	2 (3.4)	2 (4.8)	0	0	0	0
Frailty prevalence %						
# articles reporting	66	44	2	4	12	4
Range	2.4–100.0	2.4–87.5	75.6–100.0	25.0–48.0	2.9–77.0	11.1–71.0
Median (IQR)	32.8 (21.3–51.1)	31.9 (21.3–47.7)	87.8 (N/A)	34.7 (25.6–46.5)	40.25 (16.8–54.3)	44.4 (16.5–67.3)
Reason frailty measured n (%)						
Risk stratification	85 (46.4)	61 (53.0)	6 (60.0)	5 (38.5)	8 (25.0)	4 (33.3)
Inclusion/exclusion	14 (7.7)	5 (4.3)	0	1 (7.7)	6 (18.8)	2 (16.7)
Comparison with other scales	7 (3.8)	3 (2.6)	0	1 (7.7)	3 (9.4)	0
Outcome measure	9 (4.9)	6 (5.2)	0	2 (15.4)	0	1 (8.3)
Clinical decision	3 (1.6)	3 (2.6)	0	0	0	0
Descriptive only	33 (18.0)	20 (17.4)	2 (20.0)	2 (15.3)	7 (21.9)	2 (16.7)
Risk stratification and comparison of scales	21 (11.5)	11 (9.6)	2 (20.0)	1 (7.7)	5 (15.6)	2 (16.7)
Other mixed uses	10 (5.5)	6 (5.2)	0	0	0	1 (8.3)

Note that as one article did not specify setting, it is only included in the “All” column

^a^Other staff includes personnel such as paramedics, LTC staff, junior medical staff and program evaluators

# number; *IQR* interquartile range

**Fig. 3:**
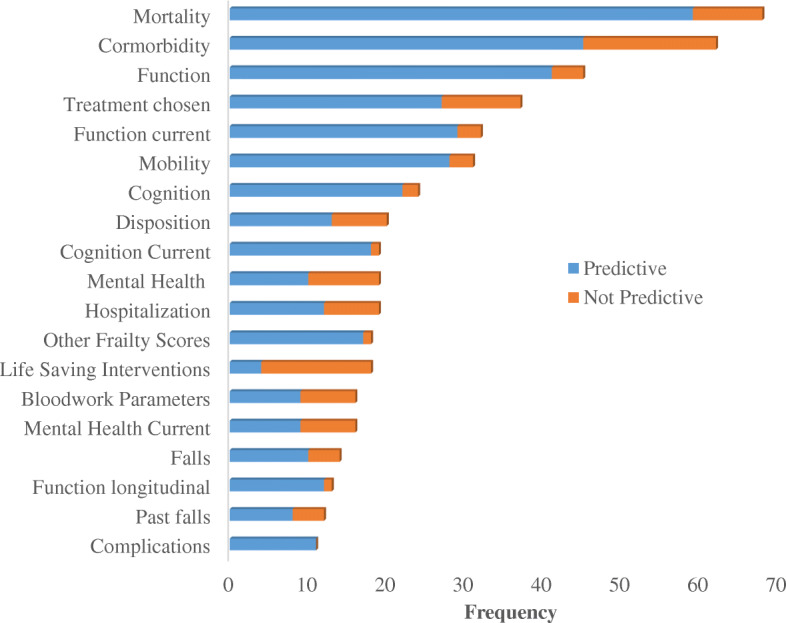
Association of CFS with most frequent outcomes. *Note that variables such as “cognition” and “function” if not otherwise specified represent combined cross-sectional and longitudinal outcomes. “Current” indicates cross-sectional outcomes only and “Longitudinal” indicates longitudinal outcomes only

The association of the CFS with outcomes was examined 526 times and was a significant predictor in 390 (74%) (Fig. 3). Several outcomes were examined, including mortality, comorbidity, disability, length of hospitalization, re-admission, institutionalization, cognitive function and falls. Mortality was the most common outcome measure, evaluated 68 times. The CFS was significantly associated with mortality in 87% of the cases. Of the 62 instances that looked at the association of CFS with comorbidity, 73% found a significant positive correlation. The CFS score was predictive of function (current function or functional decline) in 91% of the 45 times function was evaluated. The association of the CFS with the type of treatment received by patients was examined 37 times and the CFS was significantly associated in 73% of the cases. CFS score was significantly associated with mobility in 90% of the 31 instances. In addition, of the 18 studies that looked at the correlation between CFS scores and other measures of frailty 94% found significant association (Fig. 3). A number of other outcomes and variables were examined for association with the Clinical Frailty Scale in smaller numbers of studies, and these are outlined in Supplementary Table 2.

## Discussion

In this scoping review, we have reviewed the research evidence pertaining to the CFS. The CFS has been used in a variety of contexts around the world. Although most administered in Canada and the United Kingdom use of this tool has reached Asia, South America, and other parts of Europe. The number of publications measuring the CFS has increased in recent years, possibly reflecting the medical community’s interest in the topic of frailty as an important construct. The CFS is most often used in hospital settings, particularly on Geriatric Medicine and Cardiology units, however, has been applied to a range of inpatient and outpatient populations. The increase of its use in a variety of settings shows that researchers and clinicians value the ease and efficiency of using this judgement-based tool. In research, the CFS is commonly used to predict health outcomes. The outcomes with which it most has been reported to be significantly associated are mortality, comorbidity, functional decline, mobility, and cognitive decline.

This study was scoping in nature, allowing us to capture a broad range of information about the use of the CFS in research. However, this study is limited by the fact that it is not a meta-analysis and thus does not examine the quality of the evidence presented in each article. It was our aim to provide a high-level overview of this scale and its uses. In addition, the initial search was completed in 2017 and therefore does not incorporate articles published more recently.

In recent years, as interest in frailty as a medical construct has increased, so too has the number of publications measuring the CFS. Overall, these studies have assessed the CFS in a range of different populations, which as a whole are representative of the general population of older adults. Preliminary analysis of this data demonstrated that the CFS has been found to be associated with a variety of important patient characteristics and clinical outcomes. Since the CFS combines clinical judgment with objective measurement and can be easily conducted, it has been seen as one of the most promising and practical ways of screening frailty in routine assessment [11] and especially in acute care [12].

This further supports the use of the CFS as a tool in clinical practice, as it provides valuable information to guide patient care and health policy development. Synthesizing the evidence around CFS and gaining a better understanding of its feasibility and psychometric properties, could assist with the development of education materials for clinicians about how the CFS can better inform the care they provide and how it can help them discuss with patients and their families the risks and benefits of potential treatments. This will assist clinicians to provide more informed and rational shared decision making. Most recently, beyond what had been imagined when designed, and not without controversy [13–16], the CFS has been suggested as a tool that can guide rationing of critical care resources if they become overwhelmed in the COVID-19 pandemic [17]. For this reason, we published a guide for using the Clinical Frailty Scale for people new to the scale [18].

The CFS is mostly used within geriatric medicine, cardiology, intensive care, general medicine, emergency medicine, surgery, and dialysis; information for other medical specialties is lacking. The CFS is also not commonly used to predict patient-oriented measures such as quality of life, which should be included in future observation studies using the CFS. Using the CFS to assess the degree of frailty in clinical settings extends beyond evaluating risk to mitigating frailty by understanding disease presentation, acute management, recovery time, and rehabilitation potential.

Further research into the potential of this tool is warranted and will likely reveal novel applications to improve medical care of older adults. For example, investigation is warranted into whether implementing CFS in routine practice will improve care. In certain NHS centers in the United Kingdom, the CFS is routinely used to screen all patients over the age of 75 who are admitted to hospital via the Emergency Department [10]. Data from these institutions will be highly valuable in the advancement of frailty research.

## Conclusions

This scoping review revealed that the CFS has been widely used in multiple settings. Most of the included studies were conducted in hospital settings and most articles that examined the association of the CFS with adverse health outcomes showed that it has good predictive ability. The association of CFS score with clinical outcomes highlights its utility in the care of the aging population.

### Supplementary information

**Additional file 1.** Supplementary figures & tables.

## Data Availability

The datasets used and/or analyzed during the current study are available from the corresponding author on reasonable request.

## Appendix 1

### SEARCH STRATEGY

((Clinical or “Canadian Study of Health and Aging” or CSHA or Rockwood or Videx or Dalhousie or “Capital District Health Authority” or CDHA) adj2 frailty adj2 (scale* or tool* or screen* or measure* or assess* or score* or index*)).tw.

## Appendix 2

### Included Articles

1. Alfaadhel TA, Soroka SD, Kiberd BA, Landry D, Moorhouse P, Tennankore KK. Frailty and Mortality in Dialysis: Evaluation of a Clinical Frailty Scale. *Clinical Journal of the American Society of Nephrology*. 2015;10 (5):832–840. doi:10.2215/cjn.07760814.
2. Bagshaw SM, Stelfox HT, Mcdermid RC, et al. Association between frailty and short- and long-term outcomes among critically ill patients: a multicentre prospective cohort study. *Canadian Medical Association Journal*. 2013;186 (2). doi:10.1503/cmaj.130639.
3. Bagshaw SM, Stelfox HT, Johnson JA, et al. Long-Term Association Between Frailty and Health-Related Quality of Life Among Survivors of Critical Illness. *Critical Care Medicine*. 2015;43 (5):973–982. doi:10.1097/ccm.0000000000000860.
4. Bagshaw SM, Majumdar SR, Rolfson DB, Ibrahim Q, Mcdermid RC, Stelfox HT. A prospective multicenter cohort study of frailty in younger critically ill patients. *Critical Care*. 2016;20 (1). doi:10.1186/s13054-016-1338-x.
5. Baimatova I, Smith C, Beckert L, Singh H. Treatment of octogenarians with lung cancer: A single centre audit of treatments and outcomes. *New Zealand Medical Journal*. 2015;128(1419):29–34. doi:10.1016/j.hlc.2014.12.019.
6. Basic D, Shanley C. Frailty in an Older Inpatient Population. *Journal of Aging and Health*. 2014;27 (4):670–685. doi:10.1177/0898264314558202.
7. Basic D, Hartwell T. Falls in hospital and new placement in a nursing home among older people hospitalized with acute illness. *Clinical Interventions in Aging*. 2015:1637. doi:10.2147/cia.s90296.
8. Basic D, Khoo A. New medical diagnoses and length of stay of acutely unwell older patients: Implications for funding models. *Australasian Journal on Ageing*. 2015;34 (3):160–165. doi:10.1111/ajag.12160.
9. Belga S, Majumdar SR, Kahlon S, et al. Comparing three different measures of frailty in medical inpatients: Multicenter prospective cohort study examining 30-day risk of readmission or death. *Journal of Hospital Medicine*. 2016;11 (8):556–562. doi:10.1002/jhm.2607.
10. Biancari F, Ruggieri VG, Perrotti A, et al. European Multicenter Study on Coronary Artery Bypass Grafting (E-CABG registry): Study Protocol for a Prospective Clinical Registry and Proposal of Classification of Postoperative Complications. *Journal of Cardiothoracic Surgery*. 2015;10 (1). doi:10.1186/s13019-015-0292-z.
11. Borodni B, Saia F, Cuica C, et al. Prevalence of degenerative aortic stenosis in the elderly: Results of an epidemiological study of the community. *Giornale italiano di cardiologia*. 2013;14 (4):262–268.
12. Brummel NE, Jackson JC, Girard TD, et al. A Combined Early Cognitive and Physical Rehabilitation Program for People Who Are Critically Ill: The Activity and Cognitive Therapy in the Intensive Care Unit (ACT-ICU) Trial. *Physical Therapy*. 2012;92 (12):1580–1592. doi:10.2522/ptj.20110414.
13. Brummel NE, Bell SP, Girard TD, et al. Frailty and Subsequent Disability and Mortality among Patients with Critical Illness. *American Journal of Respiratory and Critical Care Medicine*. 2017;196 (1):64–72. doi:10.1164/rccm.201605-0939oc.
14. Buckstein R, Wells RA, Zhu N, et al. Patient-related factors independently impact overall survival in patients with myelodysplastic syndromes: an MDS-CAN prospective study. *British Journal of Haematology*. 2016;174 (1):88–101. doi:10.1111/bjh.14033.
15. Campo G, Pavasini R, Maietti E, et al. The frailty in elderly patients receiving cardiac interventional procedures (FRASER) program: rational and design of a multicenter prospective study. *Aging Clinical and Experimental Research*. 2016;29 (5):895–903. doi:10.1007/s40520-016-0662-y.
16. Chan D-C(D, Tsou H-H, Chen C-Y, Chen C-Y. Validation of the Chinese-Canadian study of health and aging clinical frailty scale (CSHA-CFS) telephone version. *Archives of Gerontology and Geriatrics*. 2010;50 (3). doi:10.1016/j.archger.2009.06.004.
17. Chan D-CD, Tsou H-H, Yang R-S, et al. A pilot randomized controlled trial to improve geriatric frailty. *BMC Geriatrics*. 2012;12 (1). doi:10.1186/1471-2318-12-58.
18. Chan T-C, Hung IF-N, Chan K-H, et al. Response to Comments on “Immunogenicity and Safety of Intradermal Trivalent Influenza Vaccination in Nursing Home Older Adults: A Randomized Controlled Trial.” *Journal of the American Medical Directors Association*. 2014;15 (10):773–774. doi:10.1016/j.jamda.2014.07.014.
19. Chang C-I, Chan D-C(D, Kuo K-N, Hsiung CA, Chen C-Y. Vitamin D insufficiency and frailty syndrome in older adults living in a Northern Taiwan community. *Archives of Gerontology and Geriatrics*. 2010;50. doi:10.1016/s0167-4943(10)70006-6.
20. Chang C-I, Chan D-C, Kuo K-N, Hsiung CA, Chen C-Y. Prevalence and Correlates of Geriatric Frailty in a Northern Taiwan Community. *Journal of the Formosan Medical Association*. 2011;110 (4):247–257. doi:10.1016/s0929-6646(11)60037-5.
21. Chong E, Ho E, Baldevarona-Llego J, Chan M, Wu L, Tay L. Frailty and Risk of Adverse Outcomes in Hospitalized Older Adults: A Comparison of Different Frailty Measures. *Journal of the American Medical Directors Association*. 2017;18 (7). doi:10.1016/j.jamda.2017.04.011.
22. Cockburn J, Singh MS, Rafi NHM, et al. Poor mobility predicts adverse outcome better than other frailty indices in patients undergoing transcatheter aortic valve implantation. *Catheterization and Cardiovascular Interventions*. 2015;86 (7):1271–1277. doi:10.1002/ccd.25991.
23. Coleman SA, Cunningham CJ, Walsh JB, et al. Outcomes among older people in a post-acute inpatient rehabilitation unit. *Disability and Rehabilitation*. 2011;34 (15):1333–1338. doi:10.3109/09638288.2011.636136.
24. Conroy S, Dowsing T. The ability of frailty to predict outcomes in older people attending an acute medical unit. *Acute Medicine*. 2013;12 (2):74–76.
25. Conroy S, Dowsing T, Reid J, Hsu R. Understanding readmissions: An in-depth review of 50 patients readmitted back to an acute hospital within 30 days. *European Geriatric Medicine*. 2013;4 (1):25–27. doi:10.1016/j.eurger.2012.02.007.
26. Cooksley T, Merten H, Kellett J, et al. PRISMA analysis of 30 day readmissions to a tertiary cancer hospital. *Acute Medicine*. 2015;14 (2):53–56.
27. Davis DH, Rockwood MR, Mitnitski AB, Rockwood K. Impairments in mobility and balance in relation to frailty. *Archives of Gerontology and Geriatrics*. 2011;53 (1):79–83. doi:10.1016/j.archger.2010.06.013.
28. Denvir MA, Cudmore S, Highet G, et al. Phase 2 Randomised Controlled Trial and Feasibility Study of Future Care Planning in Patients with Advanced Heart Disease. *Scientific Reports*. 2016;6 (1). doi:10.1038/srep24619.
29. Doba N, Tokuda Y, Goldstein NE, Kushiro T, Hinohara S. A pilot trial to predict frailty syndrome: The Japanese Health Research Volunteer Study. *Experimental Gerontology*. 2012;47 (8):638–643. doi:10.1016/j.exger.2012.05.016.
30. Doba N, Tokuda Y, Saiki K, et al. Assessment of Self-Efficacy and its Relationship with Frailty in the Elderly. *Internal Medicine*. 2016;55 (19):2785–2792. doi:10.2169/internalmedicine.55.6924.
31. Dólera-Moreno C, Palazón-Bru A, Colomina-Climent F, Gil-Guillén VF. Construction and internal validation of a new mortality risk score for patients admitted to the intensive care unit. *International Journal of Clinical Practice*. 2016;70 (11):916–922. doi:10.1111/ijcp.12851.
32. Eeles E, Whiting E, Tattam K, Hay K, Pandy S, Turner M. Hospitals in the home for patients with delirium: No place like home? *Australasian Medical Journal*. 2016;9 (11). doi:10.21767/amj.2016.2702.
33. Ekerstad N, Löfmark R, Andersson D, Carlsson P. A tentative consensus-based model for priority setting: An example from elderly patients with myocardial infarction and multi-morbidity. *Scandinavian Journal of Public Health*. 2011;39 (4):345–353. doi:10.1177/1403494811405092.
34. Ekerstad N, Swahn E, Janzon M, et al. Frailty Is Independently Associated With Short-Term Outcomes for Elderly Patients With Non–ST-Segment Elevation Myocardial Infarction. *Circulation*. 2011;124(22):2397–2404. doi:10.1161/circulationaha.111.025452.
35. Ekerstad N, Swahn E, Janzon M, et al. Frailty is independently associated with 1-year mortality for elderly patients with non-ST-segment elevation myocardial infarction. *European Journal of Preventive Cardiology*. 2013;21 (10):1216–1224. doi:10.1177/2047487313490257.
36. El-Sharkawy AM, Watson P, Neal KR, et al. Hydration and outcome in older patients admitted to hospital (The HOOP prospective cohort study). *Age and Ageing*. 2015;44 (6):943–947. doi:10.1093/ageing/afv119.
37. Ewan VC, Sails AD, Walls AWG, Rushton S, Newton JL. Dental and Microbiological Risk Factors for Hospital-Acquired Pneumonia in Non-Ventilated Older Patients. *Plos One*. 2015;10 (4). doi:10.1371/journal.pone.0123622.
38. Fisher C, Karalapillai DK, Bailey M, Glassford NG, Bellomo R, Jones D. Predicting Intensive Care and Hospital Outcome with the Dalhousie Clinical Frailty Scale: A Pilot Assessment. *Anaesthesia and Intensive Care*. 2015;43 (3):361–368. doi:10.1177/0310057x1504300313.
39. Forman J, Baumbusch J, Currie L, Lauck S. Exploring Changes in Functional Status While Waiting for Transcatheter Aortic Valve Implantation. *Canadian Journal of Cardiology*. 2013;29 (10). doi:10.1016/j.cjca.2013.07.721.
40. Frank C, Touw M, Suurdt J, Jiang X, Wattam P, Heyland D. Optimizing end-of-life care on medical clinical teaching units using the CANHELP Questionnaire and a Nurse Facilitator: A feasibility study. *Canadian Journal of Nursing Research*. 2012;44 (1):40–58.
41. Fukuda N, Ueno H, Hirai T, Inoue H, Kingawa K. Transcatheter aortic valve implantation in an elderly man with severe aortic stenosis and high surgical risk. *Japanese Journal of Geriatrics*. 2016;53 (2):158–163.
42. Garzon H, Restreop C, Espitia E, Torregrosa L, Dominguez LC. Fragilidad quirúrgica: un factor predictor de morbilidad y mortalidad posoperatoria en adultos mayores sometidos a cirugía abdominal de urgencia. *Revista Colombiana de Cirugía*. 2014;29 (4):278–292.
43. Gillespie D, Hood K, Bayer A, et al. Antibiotic prescribing and associated diarrhoea: a prospective cohort study of care home residents. *Age and Ageing*. 2015;44 (5):853–860. doi:10.1093/ageing/afv072.
44. Goldstein J, Hubbard RE, Moorhouse P, Andrew MK, Mitnitski A, Rockwood K. The validation of a care partner-derived frailty index based upon comprehensive geriatric assessment (CP-FI-CGA) in emergency medical services and geriatric ambulatory care. *Age and Ageing*. 2014;44 (2):327–330. doi:10.1093/ageing/afu161.
45. Gregorevic KJ, Hubbard RE, Katz B, Lim WK. The clinical frailty scale predicts functional decline and mortality when used by junior medical staff: a prospective cohort study. *BMC Geriatrics*. 2016;16 (1). doi:10.1186/s12877-016-0292-4.
46. Grossman D, Rootenberg M, Perri G-A, et al. Enhancing Communication in End-of-Life Care: A Clinical Tool Translating Between the Clinical Frailty Scale and the Palliative Performance Scale. *Journal of the American Geriatrics Society*. 2014;62 (8):1562–1567. doi:10.1111/jgs.12926.
47. Gualano B, Macedo AR, Alves CRR, et al. Creatine supplementation and resistance training in vulnerable older women: A randomized double-blind placebo-controlled clinical trial. *Experimental Gerontology*. 2014;53:7–15. doi:10.1016/j.exger.2014.02.003.
48. Hajek A, Brettschneider C, Lange C, et al. Longitudinal predictors of institutionalization in old age. *PLoS ONE*. 2015;10 (12).
49. Hajek A, Brettschneider C, Posselt T, et al. Predictors of frailty in old age–results of a longitudinal study. *The journal of nutrition, health &amp; aging*. 2015;20 (9):952–957. doi:10.1007/s12603-015-0634-5.
50. Harkness K, Heckman G, Demers C, Mckelvie R. 656 The Association Between Cognitive Function and Self-Care in Older Patients With Heart Failure. *Canadian Journal of Cardiology*. 2012;28 (5). doi:10.1016/j.cjca.2012.07.592.
51. Hartley P, Adamson J, Cunningham C, Embleton G, Romero-Ortuno R. Higher Physiotherapy Frequency Is Associated with Shorter Length of Stay and Greater Functional Recovery in Hospitalized Frail Older Adults: A Retrospective Observational Study. *The Journal of frailty &amp; aging*. 2016;5 (2):121–125.
52. Hartley P, Adamson J, Cunningham C, Embleton G, Romero-Ortuno R. Clinical frailty and functional trajectories in hospitalized older adults: A retrospective observational study. *Geriatrics &amp; Gerontology International*. 2016;17 (7):1063–1068. doi:10.1111/ggi.12827.
53. Hawker M, Romero-Ortuni R. Social Determinants of Discharge Outcomes in Older People Admitted to a Geriatric Medicine Ward. *The Journal of frailty &amp; aging*. 2016;5 (2):118–120.
54. Hayanga J, Murphy E, Girgis R, Jansma S, Khaghani A. Extracorporeal Membrane Oxygenation as a Bridge to Lung Transplantation in Patients Over Age 70 Years: A Case Report. *Transplantation Proceedings*. 2017;49 (1):218–220. doi:10.1016/j.transproceed.2016.11.025.
55. Henderson EJ, Lord SR, Close JC, Lawrence AD, Whone A, Ben-Shlomo Y. The ReSPonD trial - rivastigmine to stabilise gait in Parkinson’s disease a phase II, randomised, double blind, placebo controlled trial to evaluate the effect of rivastigmine on gait in patients with Parkinson’s disease who have fallen. *BMC Neurology*. 2013;13 (1). doi:10.1186/1471-2377-13-188.
56. Hernandez C, Aibar J, Batlle JD, et al. Assessment of health status and program performance in patients on long-term oxygen therapy. *Respiratory Medicine*. 2015;109 (4):500–509. doi:10.1016/j.rmed.2015.01.005.
57. Hewitt J, Moug SJ, Middleton M, Chakrabarti M, Stechman MJ, Mccarthy K. Prevalence of frailty and its association with mortality in general surgery. *The American Journal of Surgery*. 2015;209 (2):254–259. doi:10.1016/j.amjsurg.2014.05.022.
58. Hewitt J, Mccormack C, Tay HS, et al. Prevalence of multimorbidity and its association with outcomes in older emergency general surgical patients: an observational study. *BMJ Open*. 2016;6 (3). doi:10.1136/bmjopen-2015-010126.
59. Heyland DK, Dodek P, Mehta S, et al. Admission of the very elderly to the intensive care unit: Family members’ perspectives on clinical decision-making from a multicenter cohort study. *Palliative Medicine*. 2015;29 (4):324–335. doi:10.1177/0269216314566060.
60. Heyland D, Cook D, Bagshaw SM, et al. The Very Elderly Admitted to ICU. *Critical Care Medicine*. 2015;43 (7):1352–1360. doi:10.1097/ccm.0000000000001024.
61. Heyland DK, Ilan R, Jiang X, You JJ, Dodek P. The prevalence of medical error related to end-of-life communication in Canadian hospitals: results of a multicentre observational study. *BMJ Quality &amp; Safety*. 2015;25 (9):671–679. doi:10.1136/bmjqs-2015-004567.
62. Heyland DK, Stelfox HT, Garland A, et al. Predicting Performance Status 1 Year After Critical Illness in Patients 80 Years or Older. *Critical Care Medicine*. 2016;44 (9):1718–1726. doi:10.1097/ccm.0000000000001762.
63. Hickey A, Gleeson M, Kellett J. READS: the Rapid Electronic Assessment Documentation System. *British Journal of Nursing*. 2012;21(22):1333–1339. doi:10.12968/bjon.2012.21.22.1333.
64. Hominick K, Mcleod V, Rockwood K. Characteristics of Older Adults Admitted to Hospital versus Those Discharged Home, in Emergency Department Patients Referred to Internal Medicine. *Canadian Geriatrics Journal*. 2016;19 (1). doi:10.5770/cgj.19.195.
65. Hood K, Nuttall J, Gillespie D, et al. Probiotics for Antibiotic-Associated Diarrhoea (PAAD): a prospective observational study of antibiotic-associated diarrhoea (including *Clostridium difficile*-associated diarrhoea) in care homes. *Health Technology Assessment*. 2014;18(63):1–84. doi:10.3310/hta18630.
66. Hoogeboom TJ, Dronkers JJ, Ende CHVD, Oosting E, Meeteren NLV. Preoperative therapeutic exercise in frail elderly scheduled for total hip replacement: a randomized pilot trial. *Clinical Rehabilitation*. 2010;24 (10):901–910. doi:10.1177/0269215510371427.
67. Hoogendijk EO, Horst HEVD, Deeg DJH, et al. The identification of frail older adults in primary care: comparing the accuracy of five simple instruments. *Age and Ageing*. 2012;42 (2):262–265. doi:10.1093/ageing/afs163.
68. Hubbard RE, Andrew MK, Fallah N, Rockwood K. Comparison of the prognostic importance of diagnosed diabetes, co-morbidity and frailty in older people. *Diabetic Medicine*. 2010;27 (5):603–606. doi:10.1111/j.1464-5491.2010.02977.x.
69. Isawa T, Tada N, Ootomo T. Delayed-Onset Left Main Coronary Artery Obstruction More than 24 Hours after Balloon-Expandable Transcatheter Aortic Valve Replacement. *Texas Heart Institute Journal*. 2016;43 (5):441–445. doi:10.14503/thij-15-5475.
70. Islam A, Muir-Hunter SW, Speechley M, Montero-Odasso M. Facilitating Frailty Identification: Comparison of Two Methods among Community-Dwelling Order Adults. *The Journal of frailty &amp; aging*. 2014;3 (4):216–221.
71. Iyasere OU, Brown EA, Johansson L, et al. Quality of Life and Physical Function in Older Patients on Dialysis: A Comparison of Assisted Peritoneal Dialysis with Hemodialysis. *Clinical Journal of the American Society of Nephrology*. 2015;11 (3):423–430. doi:10.2215/cjn.01050115.
72. Jackson TA, Maclullich AMJ, Gladman JRF, Lord JM, Sheehan B. Undiagnosed long-term cognitive impairment in acutely hospitalised older medical patients with delirium: a prospective cohort study. *Age and Ageing*. 2016;45 (4):493–499. doi:10.1093/ageing/afw064.
73. Jackson TA, Moorey HC, Sheehan B, Maclullich AM, Gladman JR, Lord JM. Acetylcholinesterase Activity Measurement and Clinical Features of Delirium. *Dementia and Geriatric Cognitive Disorders*. 2016;43 (1, 2):29–37. doi:10.1159/000452832.
74. Jing Y, Shaheen E, Drake RR, Chen N, Gravenstein S, Deng Y. Aging is associated with a numerical and functional decline in plasmacytoid dendritic cells, whereas myeloid dendritic cells are relatively unaltered in human peripheral blood. *Human Immunology*. 2009;70 (10):777–784. doi:10.1016/j.humimm.2009.07.005.
75. Juma S, Taabazuing M-M, Montero-Odasso M. Clinical Frailty Scale in an Acute Medicine Unit: a Simple Tool That Predicts Length of Stay. *Canadian Geriatrics Journal*. 2016;19 (2). doi:10.5770/cgj.19.196.
76. Kahlon S, Pederson J, Majumdar SR, et al. Association between frailty and 30-day outcomes after discharge from hospital. *Canadian Medical Association Journal*. 2015;187 (11):799–804. doi:10.1503/cmaj.150100.
77. Kamo T, Nishida Y. Direct and indirect effects of nutritional status, physical function and cognitive function on activities of daily living in Japanese older adults requiring long-term care. *Geriatrics &amp; Gerontology International*. 2013;14 (4):799–805. doi:10.1111/ggi.12169.
78. Kamo T, Takayama K, Ishii H, Suzuki K, Eguchi K, Nishida Y. Coexisting severe frailty and malnutrition predict mortality among the oldest old in nursing homes: A 1-year prospective study. *Archives of Gerontology and Geriatrics*. 2017;70:99–104. doi:10.1016/j.archger.2017.01.009.
79. Kang L, Zhang SY, Zhu WL, et al. Is frailty associated with short-term outcomes for elderly patients with acute coronary syndrome? *Journal of Geriatric Cardiology*. 2015;12 (6):662–667.
80. Kellett J, Clifford M, Ridley A, Murray A, Gleeson M. A four item scale based on gait for the immediate global assessment of acutely ill medical patients – one look is more than 1000 words. *European Geriatric Medicine*. 2014;5 (2):92–96. doi:10.1016/j.eurger.2013.11.011.
81. Khadaroo RG, Padwal RS, Wagg AS, Clement F, Warkentin LM, Holroyd-Leduc J. Optimizing senior’s surgical care - Elder-friendly Approaches to the Surgical Environment (EASE) study: rationale and objectives. *BMC Health Services Research*. 2015;15 (1). doi:10.1186/s12913-015-1001-2.
82. Kleczynski P, Dziewierz A, Bagienski M, et al. Long-term mortality and quality of life after transcatheter aortic valve insertion in very elderly patients. *Journal of Invasive Cardiology*. 2016;28 (12):492–496.
83. Kleczynski P, Dziewierz A, Bagienski M, et al. Impact of frailty on mortality after transcatheter aortic valve implantation. *American Heart Journal*. 2017;185:52–58.
84. Klindtworth K, Oster P, Hager K, Krause O, Bleidorn J, Schneider N. Living with and dying from advanced heart failure: understanding the needs of older patients at the end of life. *BMC Geriatrics*. 2015;15 (1). doi:10.1186/s12877-015-0124-y.
85. Kobe AR, Meyer A, Elmubarak H, et al. Frailty Assessed by the Forecast is a Valid Tool to Predict Short-Term Outcome after Transcatheter Aortic Valve Replacement. *Innovations: Technology and Techniques in Cardiothoracic and Vascular Surgery*. 2016;11 (6):407–413. doi:10.1097/imi.0000000000000321.
86. Krikscionaitiene A, Dambrauskas Z, Barron T, Vaitkaitiene E, Vaitkaitis D. Are two or four hands needed for elderly female bystanders to achieve the required chest compression depth during dispatcher-assisted CPR: a randomized controlled trial. *Scandinavian Journal of Trauma, Resuscitation and Emergency Medicine*. 2016;24 (1). doi:10.1186/s13049-016-0238-z.
87. Lam K, Leung MF, Kwan CW, Kwan J. Severe Spastic Contractures and Diabetes Mellitus Independently Predict Subsequent Minimal Trauma Fractures Among Long-Term Care Residents. *Journal of the American Medical Directors Association*. 2016;17 (11):1025–1030. doi:10.1016/j.jamda.2016.06.029.
88. Lam P-P, Coleman BL, Green K, et al. Predictors of influenza among older adults in the emergency department. *BMC Infectious Diseases*. 2016;16 (1). doi:10.1186/s12879-016-1966-4.
89. Langford D, Ashe M, Fleig L, et al. Back to the future – feasibility of recruitment and retention to patient education and telephone follow-up after hip fracture: a pilot randomized controlled trial. *Patient Preference and Adherence*. 2015:1343. doi:10.2147/ppa.s86922.
90. Langlois F, Vu TTM, Chasse K, Dupuis G, Kergoat M-J, Bherer L. Benefits of Physical Exercise Training on Cognition and Quality of Life in Frail Older Adults. *The Journals of Gerontology Series B: Psychological Sciences and Social Sciences*. 2012;68 (3):400–404. doi:10.1093/geronb/gbs069.
91. Larouche J, Pike J, Slobogean GP, et al. Determinants of Functional Outcome in Distal Radius Fractures in High-Functioning Patients Older Than 55 Years. *Journal of Orthopaedic Trauma*. 2016;30 (8):445–449. doi:10.1097/bot.0000000000000566.
92. Lau D, Majumdar SR, Mcalister FA. Patient isolation precautions and 30-day risk of readmission or death after hospital discharge: a prospective cohort study. *International Journal of Infectious Diseases*. 2016;43:74–76. doi:10.1016/j.ijid.2015.12.018.
93. Lau D, Padwal RS, Majumdar SR, et al. Patient-Reported Discharge Readiness and 30-Day Risk of Readmission or Death: A Prospective Cohort Study. *The American Journal of Medicine*. 2016;129 (1):89–95. doi:10.1016/j.amjmed.2015.08.018.
94. Layland J, Oldroyd KG, Curzen N, et al. Fractional flow reserve vs. angiography in guiding management to optimize outcomes in non-ST-segment elevation myocardial infarction: the British Heart Foundation FAMOUS-NSTEMI randomized trial. *European Heart Journal*. 2014;36 (2):100–111. doi:10.1093/eurheartj/ehu338.
95. Leahy-Warren P, O’Caoimh R, Cochrane A, et al. Components of the Risk Instrument for Screening in the Community (RISC) that Correlate with Public Health Nurses’ Perception of Risk. *The Journal of frailty &amp; aging*. 2015;4 (3):149–154.
96. Lee J, Sirois M-J, Moore L, et al. Return to the ED and hospitalisation following minor injuries among older persons treated in the emergency department: predictors among independent seniors within 6 months. *Age and Ageing*. 2015;44 (4):624–629. doi:10.1093/ageing/afv054.
97. Lefebvre M-CD, St-Onge M, Glazer-Cavanagh M, et al. The Effect of Bleeding Risk and Frailty Status on Anticoagulation Patterns in Octogenarians With Atrial Fibrillation: The FRAIL-AF Study. *Canadian Journal of Cardiology*. 2016;32 (2):169–176. doi:10.1016/j.cjca.2015.05.012.
98. Li C-M, Chang C-I, Yu W-R, Yang W, Hsu C-C, Chen C-Y. Enhancing elderly health examination effectiveness by adding physical function evaluations and interventions. *Archives of Gerontology and Geriatrics*. 2017;70:38–43. doi:10.1016/j.archger.2016.12.009.
99. Libungan B, Hirlekar G, Albertsson P. Coronary angioplasty in octogenarians with emergent coronary syndromes: study protocol for a randomized controlled trial. *Trials*. 2014;15 (1). doi:10.1186/1745-6215-15-349.
100. Liu JY. Fear of falling in robust community-dwelling older people: results of a cross-sectional study. *Journal of Clinical Nursing*. 2014;24 (3, 4):393–405. doi:10.1111/jocn.12613.
101. Liu S, Vaillancourt C, Kasaboski A, Taljaard M. Bystander fatigue and CPR quality by older bystanders: a randomized crossover trial comparing continuous chest compressions and 30:2 compressions to ventilations. *Cjem*. 2016;18 (6):461–468. doi:10.1017/cem.2016.373.
102. Lloyd A, Kendall M, Starr JM, Murray SA. Physical, social, psychological and existential trajectories of loss and adaptation towards the end of life for older people living with frailty: a serial interview study. *BMC Geriatrics*. 2016;16 (1). doi:10.1186/s12877-016-0350-y.
103. Ma HM, Yu RHY, Woo J. Recurrent hospitalisation with pneumonia is associated with higher 1-year mortality in frail older people. *Internal Medicine Journal*. 2013;43 (11):1210–1215. doi:10.1111/imj.12258.
104. Maguet PL, Roquilly A, Lasocki S, et al. Prevalence and impact of frailty on mortality in elderly ICU patients: a prospective, multicenter, observational study. *Intensive Care Medicine*. 2014. doi:10.1007/s00134-014-3253-4.
105. Martin CM. Self-rated health: patterns in the journeys of patients with multi-morbidity and frailty. *Journal of Evaluation in Clinical Practice*. 2014;20 (6):1010–1016. doi:10.1111/jep.12133.
106. Martocchia A, Frugoni P, Indiano I, et al. Screening of frailty in elderly patients with disability by the means of Marigliano–Cacciafesta polypathology scale (MCPS) and Canadian Study of Health and Aging (CSHA) scales. *Archives of Gerontology and Geriatrics*. 2013;56 (2):339–342. doi:10.1016/j.archger.2012.11.004.
107. Masud D, Norton S, Smailes S, Shelley O, Philp B, Dziewulski P. The use of a frailty scoring system for burns in the elderly. *Burns*. 2013;39 (1):30–36. doi:10.1016/j.burns.2012.03.002.
108. Matusik P, Nowak J, Tomaszewski K, et al. Hypertension among the elderly on the basis of nursing home residents population. *Polski Przeglad Kardiologiczny*. 2010;12 (3):186–191.
109. Matusik P, Tomaszewski K, Chmielowska K, et al. Severe frailty and cognitive impairment are related to higher mortality in 12-month follow-up of nursing home residents. *Archives of Gerontology and Geriatrics*. 2012;55 (1):22–24. doi:10.1016/j.archger.2011.06.034.
110. Mccrow J, Morton M, Travers C, Harvey K, Eeles E. Associations Between Dehydration, Cognitive Impairment, and Frailty in Older Hospitalized Patients: An Exploratory Study. *Journal of Gerontological Nursing*. 2016;42 (5):19–27. doi:10.3928/00989134-20,160,201-01.
111. Mcisaac DI, Taljaard M, Bryson GL, et al. Comparative assessment of two frailty instruments for risk-stratification in elderly surgical patients: study protocol for a prospective cohort study. *BMC Anesthesiology*. 2016;16 (1). doi:10.1186/s12871-016-0276-0.
112. Mckean M, Pillans P, Scott IA. A medication review and deprescribing method for hospitalised older patients receiving multiple medications. *Internal Medicine Journal*. 2016;46 (1):35–42. doi:10.1111/imj.12906.
113. Mcnelly AS, Rawal J, Shrikrishna D, et al. An Exploratory Study of Long-Term Outcome Measures in Critical Illness Survivors. *Critical Care Medicine*. 2016;44 (6). doi:10.1097/ccm.0000000000001645.
114. Meid AD, Quinzler R, Groll A, et al. Longitudinal evaluation of medication underuse in older outpatients and its association with quality of life. *European Journal of Clinical Pharmacology*. 2016;72 (7):877–885. doi:10.1007/s00228-016-2047-8.
115. Merchant RA, Banerji S, Singh G, et al. Is Trunk Posture in Walking a Better Marker than Gait Speed in Predicting Decline in Function and Subsequent Frailty? *Journal of the American Medical Directors Association*. 2016;17 (1):65–70. doi:10.1016/j.jamda.2015.08.008.
116. Milani C, Ticinesi A, Gerritsen J, et al. Gut microbiota composition and *Clostridium difficile* infection in hospitalized elderly individuals: a metagenomic study. *Scientific Reports*. 2016;6 (1). doi:10.1038/srep25945.
117. Mlynarska A, Mlynarski R, Golba KS. Frailty syndrome in patients with heart rhythm disorders. *Geriatrics &amp; Gerontology International*. 2016;17 (9):1313–1318. doi:10.1111/ggi.12868.
118. Mlynarska A, Mlynarski R, Biernat J, Sosnowski M, Golba KS. Frailty Syndrome in Heart Failure Patients who are Receiving Cardiac Resynchronization. *Pacing and Clinical Electrophysiology*. 2016;39 (4):370–374. doi:10.1111/pace.12800.
119. Moorhouse P, Mallery LH. Palliative and Therapeutic Harmonization: A Model for Appropriate Decision-Making in Frail Older Adults. *Journal of the American Geriatrics Society*. 2012;60 (12):2326–2332. doi:10.1111/j.1532-5415.2012.04210.x.
120. Murali-Krishnan R, Iqbal J, Rowe R, et al. Impact of frailty on outcomes after percutaneous coronary intervention: a prospective cohort study. *Open Heart*. 2015;2 (1). doi:10.1136/openhrt-2015-000294.
121. Myint PK, Owen S, Pearce L, et al. The prevalence of hyperglycaemia and its relationship with mortality, readmissions and length of stay in an older acute surgical population: a multicentre study. *Postgraduate Medical Journal*. 2016;92(1091):514–519. doi:10.1136/postgradmedj-2015-133,777.
122. Noble HR, Agus A, Brazil K, et al. PAlliative Care in chronic Kidney diSease: the PACKS study—quality of life, decision making, costs and impact on carers in people managed without dialysis. *BMC Nephrology*. 2015;16 (1). doi:10.1186/s12882-015-0084-7.
123. Nolan M, Power D, Long J, Horgan F. Frailty and its association with rehabilitation outcomes in a post-acute older setting. *International Journal of Therapy and Rehabilitation*. 2016;23 (1):33–40. doi:10.12968/ijtr.2016.23.1.33.
124. North C. Comprehensive geriatric assessment of a mental health service user with safeguarding needs. *Nursing Older People*. 2016;28 (5):25–29. doi:10.7748/nop.28.5.25.s25.
125. Nouvenne A, Ticinesi A, Lauretani F, et al. The prognostic value of high-sensitivity C-reactive protein and prealbumin for short-term mortality in acutely hospitalized multimorbid elderly patients: A prospective cohort study. *The journal of nutrition, health &amp; aging*. 2015;20 (4):462–468. doi:10.1007/s12603-015-0626-5.
126. Nouvenne A, Ticinesi A, Folesani G, et al. The association of serum procalcitonin and high-sensitivity C-reactive protein with pneumonia in elderly multimorbid patients with respiratory symptoms: retrospective cohort study. *BMC Geriatrics*. 2016;16 (1). doi:10.1186/s12877-016-0192-7.
127. Omura A, Matsuda H, Minami H, et al. Early and Late Outcomes of Operation for Acute Type A Aortic Dissection in Patients Aged 80 Years and Older. *The Annals of Thoracic Surgery*. 2017;103 (1):131–138. doi:10.1016/j.athoracsur.2016.05.046.
128. Ormerod JO, Ramcharitar S. Does specific interventional risk scoring better predict mortality than comorbidity in nonagenerians undergoing coronary angioplasty? *Cardiovascular Revascularization Medicine*. 2014;15 (4):258–260. doi:10.1016/j.carrev.2014.02.008.
129. O’Caoimh R, Gao Y, Svendrovski A, et al. Screening for markers of frailty and perceived risk of adverse outcomes using the Risk Instrument for Screening in the Community (RISC). *BMC Geriatrics*. 2014;14 (1). doi:10.1186/1471-2318-14-104.
130. O’Caoimh R, Fitzgerald C, Cronin U, et al. Which Part of a Short, Global Risk Assessment, the Risk Instrument for Screening in the Community, Predicts Adverse Healthcare Outcomes? *Journal of Aging Research*. 2015;2015:1–7. doi:10.1155/2015/256414.
131. O’Caoimh R, Gao Y, Svendrovski A, et al. The Risk Instrument for Screening in the Community (RISC): a new instrument for predicting risk of adverse outcomes in community dwelling older adults. *BMC Geriatrics*. 2015;15 (1). doi:10.1186/s12877-015-0095-z.
132. Padwal R, Mcalister FA, Wood PW, et al. Telemonitoring and Protocolized Case Management for Hypertensive Community-Dwelling Seniors With Diabetes: Protocol of the TECHNOMED Randomized Controlled Trial. *JMIR Research Protocols*. 2016;5 (2). doi:10.2196/resprot.5775.
133. Parmar KR, Xiu PY, Chowdhury MR, Patel E, Cohen M. In-hospital treatment and outcomes of heart failure in specialist and non-specialist services: a retrospective cohort study in the elderly. *Open Heart*. 2015;2 (1). doi:10.1136/openhrt-2014-000095.
134. Partain NS, Subramanian M, Hodgman EI, et al. Characterizing End-of-Life Care after Geriatric Burns at a Verified Level I Burn Center. *Journal of Palliative Medicine*. 2016;19 (12):1275–1280. doi:10.1089/jpm.2016.0152.
135. Pereira MC, Miralles R, Serra E, Morros A, Palacio JR, Martinez P. Peroxidación lipídica en la membrana de los linfocitos y oxidación proteica en el suero de personas ancianas, ¿marcadores potenciales de fragilidad y dependencia? Resultados preliminares. *Revista Española de Geriatría y Gerontología*. 2016;51 (1):25–28. doi:10.1016/j.regg.2015.03.002.
136. Provencher V, Sirois M-J, Ouellet M-C, et al. Decline in Activities of Daily Living After a Visit to a Canadian Emergency Department for Minor Injuries in Independent Older Adults: Are Frail Older Adults with Cognitive Impairment at Greater Risk? *Journal of the American Geriatrics Society*. 2015;63 (5):860–868. doi:10.1111/jgs.13389.
137. Provencher V, Sirois M-J, Émond M, et al. Frail older adults with minor fractures show lower health-related quality of life (SF-12) scores up to six months following emergency department discharge. *Health and Quality of Life Outcomes*. 2016;14 (1). doi:10.1186/s12955-016-0441-7.
138. Pugh J, Aggett J, Goodland A, et al. Frailty and comorbidity are independent predictors of outcome in patients referred for pre-dialysis education. *Clinical Kidney Journal*. 2016;9 (2):324–329. doi:10.1093/ckj/sfv150.
139. Puthecary Z, Mcnelly A, Rawal J, et al. Clinical frailty, subjective and objective measures in intensive care survivors. *American Journal of Respiratory and Critical Care Medicine*. 2014;189.
140. Ritt M, Bollheimer L, Sieber C, Gaßmann K. Prediction of one-year mortality by five different frailty instruments: A comparative study in hospitalized geriatric patients. *Archives of Gerontology and Geriatrics*. 2016;66:66–72. doi:10.1016/j.archger.2016.05.004.
141. Ritt M, Schülein S, Lubrich H, Bollheimer LC, Sieber CC, Gassmann K-G. High-technology based gait assessment in frail people: Associations between spatio-temporal and three-dimensional gait characteristics with frailty status across four different frailty measures. *The journal of nutrition, health &amp; aging*. 2016;21 (3):346–353. doi:10.1007/s12603-016-0764-4.
142. Ritt M, Ritt J, Sieber C, Gaßmann K. Comparing the predictive accuracy of frailty, comorbidity, and disability for mortality: a 1-year follow-up in patients hospitalized in geriatric wards. *Clinical Interventions in Aging*. 2017;Volume 12:293–304. doi:10.2147/cia.s124342.
143. Rockwood K, Abeysundera MJ, Mitnitski A. How should we grade frailty in nursing home patients? *Journal of the American Medical Directors Association*. 2007;8 (9):595–603. doi:10.1016/j.jamda.2007.07.012.
144. Rockwood K, Rockwood MRH, Mitnitski A. Physiological Redundancy in Older Adults in Relation to the Change with Age in the Slope of a Frailty Index. *Journal of the American Geriatrics Society*. 2010;58 (2):318–323. doi:10.1111/j.1532-5415.2009.02667.x.
145. Rodgers G. Applying comprehensive geriatric assessment to investigate falls. *Nursing Older People*. 2016;28 (3):27–31. doi:10.7748/nop.28.3.27.s24.
146. Romero-Ortuno R, Wallis S, Biram R, Keevil V. Clinical frailty adds to acute illness severity in predicting mortality in hospitalized older adults: An observational study. *European Journal of Internal Medicine*. 2016;35:24–34. doi:10.1016/j.ejim.2016.08.033.
147. Rosenberg T. Acute Hospital Use, Nursing Home Placement, and Mortality in a Frail Community-Dwelling Cohort Managed with Primary Integrated Interdisciplinary Elder Care at Home. *Journal of the American Geriatrics Society*. 2012;60 (7):1340–1346. doi:10.1111/j.1532-5415.2012.03965.x.
148. Sabin J, Khan W, Subbe CP, et al. ‘The time it takes … ’ How doctors spend their time admitting a patient during the acute medical take. *Clinical Medicine*. 2016;16 (4):320–324. doi:10.7861/clinmedicine.16-4-320.
149. Schneeberg A, Bettinger JA, Mcneil S, et al. Knowledge, attitudes, beliefs and behaviours of older adults about pneumococcal immunization, a Public Health Agency of Canada/Canadian Institutes of Health Research Influenza Research Network (PCIRN) investigation. *BMC Public Health*. 2014;14 (1). doi:10.1186/1471-2458-14-442.
150. Schreier MM, Bauer U, Osterbrink J, Niebauer J, Iglseder B, Reiss J. Fitness training for the old and frail. *Zeitschrift für Gerontologie und Geriatrie*. 2015;49 (2):107–114. doi:10.1007/s00391-015-0966-0.
151. Shiraishi T. Relationships between Frailty and Outcomes Following to Emergency Transportation in Elderly Patients in Rural Area. *The Kitakanto Medical Journal The KITAKANTO Medical Journal*. 2015;65 (1):39–44. doi:10.2974/kmj.65.39.
152. Shulman MA, Myles PS, Chan MTV, Mcilroy DR, Wallace S, Ponsford J. Measurement of Disability-free Survival after Surgery. *Anesthesiology*. 2015;122 (3):524–536. doi:10.1097/aln.0000000000000586.
153. Sieffert M, Sinning JM, Meyer A, et al. Development of a risk score for outcome after transcatheter aortic valve implantation. *Clinical Research in Cardiology*. 2014;103 (8):631–640.
154. Sirois M-J, Griffith L, Perry J, et al. Measuring Frailty Can Help Emergency Departments Identify Independent Seniors at Risk of Functional Decline After Minor Injuries. *The Journals of Gerontology Series A: Biological Sciences and Medical Sciences*. 2015;72 (1):68–74. doi:10.1093/gerona/glv152.
155. Solaligue DES, Hederman L, Martin CM. What weekday? How acute? An analysis of reported planned and unplanned GP visits by older multi-morbid patients in the Patient Journey Record System database. *Journal of Evaluation in Clinical Practice*. 2014;20 (4):522–526. doi:10.1111/jep.12171.
156. Springer J, Bailey J, Davis P, Johnson P. Management and outcomes of small bowel obstruction in older adult patients: a prospective cohort study. *Canadian Journal of Surgery*. 2014;57 (6):379–384. doi:10.1503/cjs.029513.
157. Stammers AN, Kehler DS, Afilalo J, et al. Protocol for the PREHAB study--Pre-operative Rehabilitation for reduction of Hospitalization After coronary Bypass and valvular surgery: a randomised controlled trial. *BMJ Open*. 2015;5 (3). doi:10.1136/bmjopen-2014-007250.
158. Subbe C, Kellett J, Whitaker C, et al. A pragmatic triage system to reduce length of stay in medical emergency admission: Feasibility study and health economic analysis. *European Journal of Internal Medicine*. 2014;25 (9):815–820. doi:10.1016/j.ejim.2014.06.001.
159. Subbe CP, Burford C, Jeune IL, Masterton-Smith C, Ward D. Relationship between input and output in acute medicine – secondary analysis of the Society for Acute Medicines benchmarking audit 2013 (SAMBA ‘13). *Clinical Medicine*. 2015;15 (1):15–19. doi:10.7861/clinmedicine.15-1-15.
160. Sundermann S, Dademasch A, Rastan A, et al. One-year follow-up of patients undergoing elective cardiac surgery assessed with the Comprehensive Assessment of Frailty test and its simplified form. *Interactive CardioVascular and Thoracic Surgery*. 2011;13 (2):119–123. doi:10.1510/icvts.2010.251884.
161. Sutorius FL, Hoogendijk EO, Prins BAH, Hout HPJV. Comparison of 10 single and stepped methods to identify frail older persons in primary care: diagnostic and prognostic accuracy. *BMC Family Practice*. 2016;17 (1). doi:10.1186/s12875-016-0487-y.
162. Sze S, Zhang J, Pellicori P, Morgan D, Hoye A, Clark AL. Prognostic value of simple frailty and malnutrition screening tools in patients with acute heart failure due to left ventricular systolic dysfunction. *Clinical Research in Cardiology*. 2017;106 (7):533–541. doi:10.1007/s00392-017-1082-5.
163. Sönnichsen A, Trampisch US, Rieckert A, et al. Polypharmacy in chronic diseases–Reduction of Inappropriate Medication and Adverse drug events in older populations by electronic Decision Support (PRIMA-eDS): study protocol for a randomized controlled trial. *Trials*. 2016;17 (1). doi:10.1186/s13063-016-1177-8.
164. Sündermann S, Dademasch A, Praetorius J, et al. Comprehensive assessment of frailty for elderly high-risk patients undergoing cardiac surgery☆. *European Journal of Cardio-Thoracic Surgery*. 2011;39 (1):33–37. doi:10.1016/j.ejcts.2010.04.013.
165. Tandon P, Tangri N, Thomas L, et al. A Rapid Bedside Screen to Predict Unplanned Hospitalization and Death in Outpatients with Cirrhosis: A Prospective Study of the Clinical Frailty Scale. *Journal of Hepatology*. 2016;64 (2). doi:10.1016/s0168-8278(16)00341-x.
166. Theou O, Brothers TD, Mitnitski A, Rockwood K. Operationalization of Frailty Using Eight Commonly Used Scales and Comparison of Their Ability to Predict All-Cause Mortality. *Journal of the American Geriatrics Society*. 2013;61 (9):1537–1551. doi:10.1111/jgs.12420.
167. Ticinesi A, Lauretani F, Nouvenne A, et al. Lung ultrasound and chest x-ray for detecting pneumonia in an acute geriatric ward. *Medicine*. 2016;95(27). doi:10.1097/md.0000000000004153.
168. Vaillancourt C, Midzic I, Taljaard M, Chisamore B. Performer fatigue and CPR quality comparing 30:2 to 15:2 compression to ventilation ratios in older bystanders: A randomized crossover trial. *Resuscitation*. 2011;82 (1):51–56. doi:10.1016/j.resuscitation.2010.09.003.
169. Villanyi D, Fok M, Wong RY. Medication Reconciliation: Identifying Medication Discrepancies in Acutely Ill Hospitalized Older Adults. *The American Journal of Geriatric Pharmacotherapy*. 2011;9 (5):339–344. doi:10.1016/j.amjopharm.2011.07.005.
170. Vogler CM, Sherrington C, Ogle SJ, Lord SR. Reducing Risk of Falling in Older People Discharged From Hospital: A Randomized Controlled Trial Comparing Seated Exercises, Weight-Bearing Exercises, and Social Visits. *Archives of Physical Medicine and Rehabilitation*. 2009;90 (8):1317–1324. doi:10.1016/j.apmr.2009.01.030.
171. Vogler CM, Menant JC, Sherrington C, Ogle SJ, Lord SR. Evidence of Detraining After 12-Week Home-Based Exercise Programs Designed to Reduce Fall-Risk Factors in Older People Recently Discharged From Hospital. *Archives of Physical Medicine and Rehabilitation*. 2012;93 (10):1685–1691. doi:10.1016/j.apmr.2012.03.033.
172. Wallis S, Wall J, Biram R, Romero-Ortuno R. Association of the clinical frailty scale with hospital outcomes. *Qjm*. 2015;108 (12):943–949. doi:10.1093/qjmed/hcv066.
173. Wang C-J, Hung C-H, Tang T-C, et al. Urinary Incontinence and Its Association with Frailty Among Men Aged 80 Years or Older in Taiwan: A Cross-Sectional Study. *Rejuvenation Research*. 2017;20 (2):111–117. doi:10.1089/rej.2016.1855.
174. Warmoth K, Lang IA, Phoenix C, et al. ‘Thinking youre old and frail’: a qualitative study of frailty in older adults. *Ageing and Society*. 2015;36 (7):1483–1500. doi:10.1017/s0144686x1500046x.
175. Wearn C, Hardwicke J, Kitsios A, Siddons V, Nightingale P, Moiemen N. Outcomes of burns in the elderly: Revised estimates from the Birmingham Burn Centre. *Burns*. 2015;41 (6):1161–1168. doi:10.1016/j.burns.2015.04.008.
176. Whiting CC, Siebert J, Newman AM, et al. Large-Scale and Comprehensive Immune Profiling and Functional Analysis of Normal Human Aging. *Plos One*. 2015;10 (7). doi:10.1371/journal.pone.0133627.
177. Wong RY, Miller WC. Adverse outcomes following hospitalization in acutely ill older patients. *BMC Geriatrics*. 2008;8 (1). doi:10.1186/1471-2318-8-10.
178. Woo J, Leung J, Morley JE. Comparison of Frailty Indicators Based on Clinical Phenotype and the Multiple Deficit Approach in Predicting Mortality and Physical Limitation. *Journal of the American Geriatrics Society*. 2012;60 (8):1478–1486. doi:10.1111/j.1532-5415.2012.04074.x.
179. Wu T-Y, Chie W-C, Kuo K-L, et al. Quality of life (QOL) among community dwelling older people in Taiwan measured by the CASP-19, an index to capture QOL in old age. *Archives of Gerontology and Geriatrics*. 2013;57 (2):143–150. doi:10.1016/j.archger.2013.03.010.
180. Wu T-Y, Chie W-C, Yang R-S, Kuo K-L, Wong W-K, Liaw C-K. Risk factors for single and recurrent falls: A prospective study of falls in community dwelling seniors without cognitive impairment. *Preventive Medicine*. 2013;57 (5):511–517. doi:10.1016/j.ypmed.2013.07.012.
181. Xi H, Shi J, Meng L, et al. [Application of frailty index for comprehensive geriatric assessment in the elderly in China]. *Journal of Saffron Epidemiology*. 2016;37 (5):718–721.
182. Yiu CJ, Khan SU, Subbe CP, Tofeec K, Madge RA. Into the night: Factors affecting response to abnormal Early Warning Scores out-of-hours and implications for service improvement. *Acute Medicine*. 13 (2):56–60.
183. You JJ, Dodek P, Lamontagne F, et al. What really matters in end-of-life discussions? Perspectives of patients in hospital with serious illness and their families. *Canadian Medical Association Journal*. 2014;186 (18). doi:10.1503/cmaj.140673.

## Abbreviation

CFS: Clinical Frailty Scale
