# Supplementary material for: A scoping review of the Clinical Frailty Scale
Source: BMC Geriatr. 2020 Oct 7;20:393. doi: 10.1186/s12877-020-01801-7 (PMC7540438; doi:10.1186/s12877-020-01801-7)
Supplement: Supplementary file 1 — Additional file 1. Supplementary figures & tables. [file 12877_2020_1801_MOESM1_ESM.docx]

**Supplementary Table 1.** Association of the CFS score with demographic variables

| Demographics | Times Variable Recorded n | Times CFS Associated n | Predictive % |
| --- | --- | --- | --- |
| Age | 31 | 24 | 77.4 |
| Female Sex | 18 | 9 | 50.0 |
| Education Level | 4 | 2 | 50.0 |
| Race | 3 | 0 | 0.0 |
| Living Alone | 3 | 3 | 100.0 |
| Marital Status | 2 | 1 | 50.0 |
| Social Isolation | 2 | 1 | 50.0 |
| Smoking | 1 | 0 | 0.0 |
| Down Syndrome | 1 | 1 | 100.0 |
| Degree of Intellectual Disability | 1 | 1 | 100.0 |
| “Health and Social Engagement” | 1 | 1 | 100.0 |

**Supplementary Table 2.** Association of CFS score with less frequent outcomes.

| Variable/Outcome | Times Outcome Measured n | Times CFS Predictive n | Percent Predictive % |
| --- | --- | --- | --- |
| Institutionalization | 10 | 7 | 70.0 |
| Quality of Life | 8 | 6 | 62.5 |
| Heart Disease | 8 | 5 | 62.5 |
| Prognostic Scales | 8 | 3 | 37.5 |
| Acuity | 7 | 3 | 42.9 |
| Nutrition | 7 | 3 | 42.9 |
| Composite Scale | 6 | 5 | 83.3 |
| CBC Parameters | 6 | 6 | 100.0 |
| Metabolic Risk Factors | 6 | 0 | 0.0 |
| Health Care Usage Overall | 5 | 4 | 80.0 |
| Cognition Longitudinal | 5 | 4 | 80.0 |
| Sensory Deficits | 4 | 4 | 100.0 |
| Bowel/Bladder Dysfunction | 4 | 4 | 100.0 |
| Infection | 4 | 0 | 0.0 |
| Health Care Usage Longitudinal | 3 | 3 | 100.0 |
| Pain | 3 | 2 | 66.7 |
| Inflammation Markers | 3 | 3 | 100.0 |
| Treatment Outcome | 3 | 2 | 66.7 |
| Care Planning | 3 | 2 | 66.7 |
| Mental Health Longitudinal | 3 | 1 | 33.3 |
| Health Care Usage Cross-sectional | 2 | 1 | 50.0 |
| Imaging | 2 | 1 | 50.0 |
| Liver Scale Scores | 2 | 1 | 50.0 |
| Cardiac Scale Scores | 2 | 2 | 100.0 |

Note. More frequent outcomes are reported in figure 4.

**Supplementary Figure 1.** Version of CFS used by year of publication

**Supplementary Figure 2.** Reason for frailty assessment, by study design

**Supplementary Figure 1.** Version of CFS used by year of publication

**Supplementary Figure 2.** Reason for frailty assessment, by study design
